# Causes of death among females–investigating beyond maternal causes: a community-based longitudinal study

**DOI:** 10.1186/1756-0500-7-629

**Published:** 2014-09-10

**Authors:** Yohannes Adama Melaku, Berhe Weldearegawi, Alemseged Aregay, Fisaha Haile Tesfay, Loko Abreha, Semaw Ferede Abera, Afework Mulugeta Bezabih

**Affiliations:** Department of Public Health, College of Health Sciences, Mekelle University, Mekelle, Ethiopia; Department of Pediatrics and Child Health, College of Health Sciences, Mekelle University, Mekelle, Ethiopia

**Keywords:** Female mortality, Cause specific mortality rate, Northern Ethiopia, Verbal autopsy

## Abstract

**Background:**

In developing countries, investigating mortality levels and causes of death among all age female population despite the childhood and maternal related deaths is important to design appropriate and tailored interventions and to improve survival of female residents.

**Methods:**

Under Kilite-Awlealo Health and Demographic Surveillance System, we investigated mortality rates and causes of death in a cohort of female population from 1st of January 2010 to 31st of December 2012. At the baseline, 33,688 females were involved for the prospective follow-up study. Households under the study were updated every six months by fulltime surveillance data collectors to identify vital events, including deaths. Verbal Autopsy (VA) data were collected by separate trained data collectors for all identified deaths in the surveillance site. Trained physicians assigned underlining causes of death using the 10th edition of International Classification of Diseases (ICD). We assessed overall, age- and cause-specific mortality rates per 1000 person-years. Causes of death among all deceased females and by age groups were ranked based on cause specific mortality rates. Analysis was performed using Stata Version 11.1.

**Results:**

During the follow-up period, 105,793.9 person-years of observation were generated, and 398 female deaths were recorded. This gave an overall mortality rate of 3.76 (95% confidence interval (CI): 3.41, 4.15) per 1,000 person-years. The top three broad causes of death were infectious and parasitic diseases (1.40 deaths per 1000 person-years), non-communicable diseases (0.98 deaths per 1000 person-years) and external causes (0.36 per 1000 person-years). Most deaths among reproductive age female were caused by Human Deficiency Virus/Acquired Immune Deficiency Virus (HIV/AIDS) and tuberculosis (0.14 per 1000 person-years for each cause). Pregnancy and childbirth related causes were responsible for few deaths among women of reproductive age—3 out of 73 deaths (4.1%) or 5.34 deaths per 1,000 person-years.

**Conclusions:**

Communicable diseases are continued to be the leading causes of death among all age females. HIV/AIDS and tuberculosis were major causes of death among women of reproductive age. Together with existing efforts to prevent pregnancy and childbirth related deaths, public health and curative interventions on other causes, particularly on HIV/AIDS and tuberculosis, should be strengthened.

## Background

Studies in sub-Saharan African countries have demonstrated a significant reduction of all-causes and Communicable diseases (CDs) related mortality among women [1–4]. Most female mortality is reportedly due to maternal causes and sexual and reproductive health related threats [1, 5, 6].

Ethiopia is the country with high rates of maternal deaths [1, 7]. However, according to 2011 report of Ethiopian health profile, life expectancy of women was higher (61.8 years) than men (59 years) [8]. One of the reasons for this could be high emphasis by the government of Ethiopia given to maternal health resulting in expansion of maternal health care services to prevent pregnancy and childbirth related deaths among women of reproductive age [9]. Despite this effort, little is known about what and in what extent other causes of death are contributing for mortality of females. Some existing studies reported that CDs are still the leading causes of death among females–with notes of increasing burden of Non-Communicable Diseases (NCDs) and injuries in the country [3, 10, 11].

For health interventions to be effective and tailored to the appropriate group, it is important for policy makers and planners to be aware of what causes death among the group [12]. In this regard, mortality data are important indicators of community health and are indispensible inputs in setting priorities for health interventions. Moreover, mortality data are crucial to develop and evaluate effective health interventions and policies [13]. However, many developing countries, including Ethiopia, with the highest burden of diseases continue to lack routine, representative and high quality information on causes of death [12, 14, 15]. Studies have shown that VA is the best available approach in obtaining evidences on causes of death in resource limited setting [16, 17].

In this regard, Health and Demographic Surveillance System (HDSS) using validated VA procedures can be used as an alternative method for ascertaining causes of death out of health facilities [18, 19]. As a result, VA method has been an epidemiological tool for some decades to estimate cause specific mortalities in a community [19, 20]. Thus, Kilite-Awlaelo Health and Demographic Surveillance System (KA-HDSS) in Tigray region has been collecting vital events and VA data since September 2009.

This study investigated causes of death among all age female residents in KA-HDSS. The study will have important policy implications by providing tailored evidences on the most common causes of death among females. It also enables decision makers and planners to allocate appropriate resources to prevent the leading causes of death among this segment of the population. Furthermore, the findings of this study will guide existing maternal health policy and strategies by providing important information on cause specific mortalities among women of reproductive age.

## Methods

Description of Study setting (including map of the study area), detail VA method and field operation system of KA-HDSS are presented elsewhere [3, 21]. In these two previous publications, mortality and VA data collection procedures and interpretation of VA data are described exhaustively.

Under KA-HDSS, we investigated mortality rates and causes of death in a cohort of female population from 1st of January 2010 to 31st of December 2012. At the baseline, 33,688 females were involved for the prospective follow-up study. Households of the residents were updated every six months by fulltime surveillance data collectors to identify vital events, including deaths.

The enumerators detected deaths in each household during which they record the name of deceased, place of death and unique identification number offered by the surveillance study. All these deaths were reported to the study supervisors for further interview using VA questionnaire. Parents or spouses are identified as respondents for VA interview. VA interviews were performed at least 45 days after the death considering the local mourning period. The surveillance system employed standard data collection tools and procedures adopted from the International Network of demographic Evaluation of people and Their Health (INDEPTH) Network [22].

### Review of verbal autopsy data and classification of diseases

The causes of death were coded based on the ICD-10 [23]. Two blinded physicians independently reviewed the completed VA questionnaires to assign cause of death using the manual. A Surveillance team member, who was in charge of this specific task, confirmed agreement between the two physicians. When disagreements in diagnosis happened, a third physician was assigned to review the case. The final diagnosis was decided based on the agreement between the third physician and any of the two physicians. The case was considered as “indeterminate” if all three physicians assigned a different diagnosis. Physician gave a diagnosis “unspecified causes of death (VA-99)” for a case when difficulties to classify diseases based on the given information were present.

After careful review of the ICD-10 [23] and the 2006 Global Burden of Diseases and risk factors [24], we defined NCDs operationally. NCDs were defined as diseases that were non-infectious, non-external causes, non-pregnancy and child birth related and non-perinatal causes. Based on this definition, NCDs included gastrointestinal disorders, respiratory disorders, mental and nervous system disorders, renal disorders, neoplasm, diseases of the circulatory system and nutrition and endocrine disorders.

***CDs (VA-01):*** all infectious and parasitic diseases (VA-01) including HIV, TB, malaria, intestinal infection, infectious diseases of unspecified cause, Acute lower respiratory infections, meningitis, viral hepatitis and typhoid and paratyphoid.

***NCDs:*** included diseases of circulatory system (VA-04), neoplasm (VA-02), renal disorders (VA-07), respiratory disorders (VA-05), gastrointestinal disorders (VA-06), mental and nervous system disorders (VA-08) and nutritional and endocrine disorders (VA-03).

***External causes of death (ECs) (VA-11)***: Accidental fall, accidental drowning and submersion, intentional self harm, assault and others which are not related to the above two categories.

***Pregnancy, childbirth and pueprium related deaths (VA-09):*** Included all deaths related to pregnancy and childbirths. For example deaths associated with abortion, childbirth related hemorrhage.

### Data management and analysis

The KA-HDSS uses Household Registration System (HRS version 2.1) FoxPro database (Microsoft Corp., Redmond, United States of America). The analysis was performed using Stata version 11.1 (Stata Corp., College Station, USA). We calculated crude mortality rates (total as well as age- and cause-specific) using the number of deaths as numerator and the time period at risk contributed by each individual in the study population as denominator. After physicians assigned probable causes of death, VA data with final diagnosis of cause of death were entered using SPSS version 16.0 and exported to Stata version 11.1. The exported VA data were merged with the surveillance data using the unique individual identification number of the deceased person. We assessed the number of deaths and the mortality rates per 1000 person-years for specific and broad categories of causes of death. Causes of death among all deceased females and by age groups were ranked based on cause specific mortality rates.

### Ethics statement

The KA-HDSS received ethical clearance from the Ethiopian Science and Technology Agency with identification number IERC-0030. Informed verbal consent was obtained from head of the family or eligible adult among the family. This verbal consent was documented in English and local language “Tigrenga”. This documentation was done by marking “Yes” or “No” for a question “Are you willing to participate in this study?” after explaining all information about confidentiality, privacy and the right to not participate or withdraw from the study. The interview will be continued if a study participant answered only the response “Yes”. This process was done for each of the study participant. All individuals who are undergone in this study had knowledge on verbal consent form and the study. To keep confidentiality, data containing personal identifiers of subjects were not shared to any third parties. All these processes were approved by the above aforementioned institution.

## Results

A total of 398 female deaths were recorded in the follow-up period. Of these, complete VA data were collected for 377 deaths. Due to incomplete nature of VA data, shortage of information or other reasons, causes were not assigned for 21 (5.3%) deaths, which were labeled as “missing” causes of death.

There were 105793.9 person-years of observation during the 36 months of the follow-up. Almost 85% of the total person-years were contributed by rural residents. Women of reproductive age (15–49 years) shared just more than half (53.1%) of the person-years observations. Almost nine out of ten, (356; 89.4%), of deaths were occurred out of health institutions (Table 1).

**Table 1:** **Socio-demographic characteristics of females in Kilite-Awlaelo health and demographic surveillance system, northern Ethiopia, 2010 -2012**

		Total (105793.9 person-years)	No. of deaths (Total = 398)	Overall crude death rate = 3.76 per 1000 person-years
Variable	Category	Person-years (%)	n	Rate/1000 person-years (95% CI)
**Residence**	Rural	89612.4 (84.7)	364	4.06 (3.67, 4.50)
	Urban	16181.4 (15.3)	34	2.10 (1.50, 2.94)
**Age category**	<1 year	464.6 (0.4)	47	101.16 (76.00, 134.64)
	1-4 years	4276.8 (4.0)	19	4.44 (2.83, 6.97)
	5-14 years	29707.1 (28.1)	24	0.81 (0.54, 1.21)
	15-49 years	56137.4 (53.1)	73	1.30 (1.03, 1.64)
	50-64 years	9758.3 (9.2)	61	6.25 (4.86, 8.03)
	65-84 years	5075.2 (4.8)	134	26.40 (22.30, 31.27)
	85+ years	374.4 (0.4)	40	106.84 (78.36, 145.64)
**Median (IQR)**	19 (IQR = 10,33)			
**Education**	illiterate	40015.4 (37.8)	284	7.10 (6.32, 7.97)
	primary education	33769.4 (31.9)	26	0.77 (0.52, 1.13)
	secondary education and above	10317.5 (9.8)	10	0.97 (0.52, 1.80)
	Unknown/missing	7687.2 (7.3)	0	0.00
	Illegible (age < 7 years)	14004.4 (13.2)	78	5.57 (4.46,6.95)
**Marital statutes**	Married	28458.3 (26.9)	82	2.88 (2.32,3.58)
	Single	34551.3 (32.7)	53	1.53 (1.17, 2.01)
	Dissolved	11989.1 (11.3)	176	14.68 (12.66, 17.02)
	Unknown	8520.8 (8.1)	5	0.59 (0.24, 0.14)
	Illegible (age < 10 years)	22274.9 (21.1)	82	3.68 (2.97, 4.57)
		**No. (%)**		
**Place of death**	Home	334 (83.9)		
	Health institution	42 (10.6)		
	Other	22 (5.5)		
	Total	398		

CI-confidence interval.

### Broad causes of death

**Figure 1:**
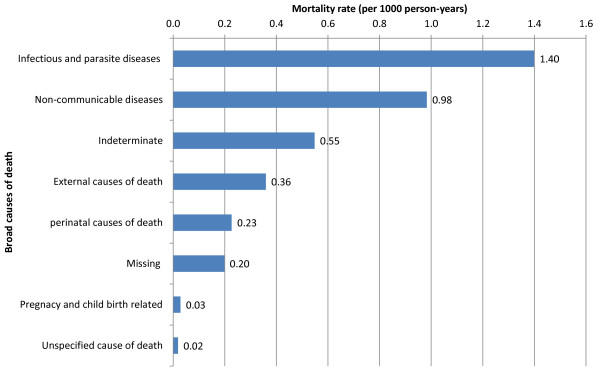
**Broad cause mortality rates (per 1000 person-years) among females in Kilite-Awlaelo Health and Demographic Surveillance System, in northern Ethiopia, 2010–2012.**

**Figure 2:**
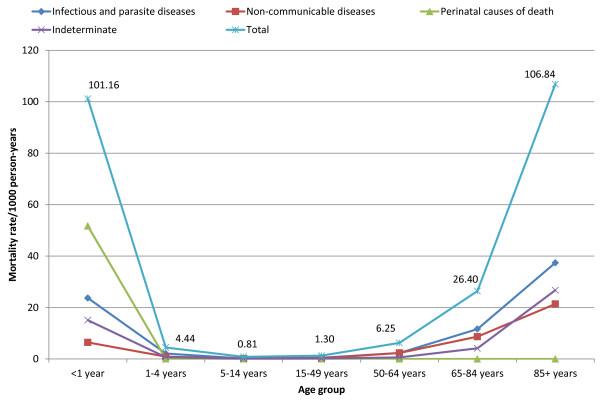
**Age-specific broad cause mortality rates (per 1000 person-years) among females in Kilite-Awlaelo Health and Demographic Surveillance System, in northern Ethiopia, 2010–2012.**

**Figure 3:**
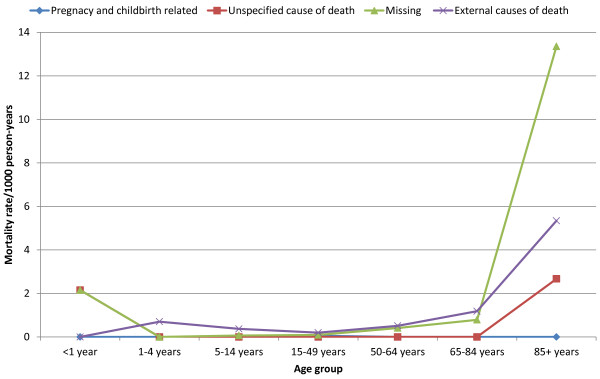
**Age-specific broad cause mortality rates (per 1000 person-years) among females in Kilite-Awlaelo Health and Demographic Surveillance System, in northern Ethiopia, 2010–2012.**

Overall mortality among female population in the study period was 3.76 (95% CI: 3.41, 4.15) per 1000 person-years. CDs were the leading causes of death accounting for 148 of 398 deaths (37.2%) or 1.40 deaths per 1000 person-years. NCDs attributed deaths were the second contributing for 104 out of 398 deaths (26.1%) or 0.98 deaths per 1000 person-years. Of all female deaths, pregnancy and childbirth related causes were responsible for few deaths—only 3 of 398 deaths (0.8%) (0.03 deaths per 1000 person-years) (Figure 1).Death rates among old age group females (85 years and above) and infants were 106.84 and 101.16 per 1000 person-years, respectively. The mortality rate decreased among early childhoods, adolescents and late adulthoods which were again increased in older age groups. The leading causes among infants were perinatal causes (51.66 deaths per 1000 person-years) followed by CDs (23.68 deaths per 1000 person-years). Whereas, the leading causes of death among the age group of 85 years and above were CDs attributing for 37.39 deaths per 1000 person-years. In this age group, NCDs were the second leading causes of death causing 21.37 deaths per 1000 person-years; and indeterminate cause of death accounted for 26.71 deaths per 1000 person-years (Figure 2 and Figure 3).

### Specific causes of death

Of all specific causes of death, tuberculosis, acute lower respiratory tract infections and intestinal infectious diseases were the top three diseases which caused majority of deaths. ECs of death were responsible for 38 (9.6%) of the deaths, or 0.36 deaths per 1000 person-years. Majority of ECs of death were due to accidental fall and accidental drowning and submersion. Of all causes, diseases of the circulatory system and perinatal causes were attributed for 37 deaths (9.3%) (0.35 deaths per 1000 person-years) and 24 (6.0%) (0.23 deaths per 1000 person-years), respectively. Our surveillance detected only 10 (0.09 per 1000 person-years) and 8 (0.08 per 1000 person-years) female deaths due to HIV/AIDS and malaria (Table 2).

**Table 2:** **Cause-specific mortality rates (per 1000 person-years) among females in Kilite-Awlaelo health and demographic surveillance system, northern Ethiopia, 2010- 2012**

VA-title	VA-code	Total (105793.9 person-years)		Rank
		n (total = 398)	Rate	
**Infectious and parasite diseases**	**VA-01**	**148**	**1.40**	
Tuberculosis	VA-01.03	61	0.58	1
Acute lower respiratory infections	VA-01.13	20	0.19	2
Intestinal infection diseases	VA-01.01	17	0.16	3
Infectious diseases, unspecified cause	VA-01.99	16	0.15	4
Meningitis	VA-01.11	14	0.13	5
HIV/AIDS	VA-01.09	10	0.09	8
Malaria	VA-01.10	8	0.08	9
Viral hepatitis	VA-01.08	2	0.02	
**External causes of death**	**VA-11**	**38**	**0.36**	
Accidental Fall	VA-11.03	13	0.12	6
Accidental drowning and submersion	VA-11.04	8	0.08	9
Others	VA-11.01, VA-11.07, VA-11.09, VA-11.11	4	0.04	
Intentional Self Harm	VA-11.10	3	0.03	
Contact with Venomous	VA-11.06	3	0.03	
Accidental Exposure to smoke, fire and flame	VA-11.05	3	0.03	
Accident unspecified	VA-11.97	2	0.02	
Other transport accident	VA-11.02	2	0.02	
**Diseases of the circulatory system**	**VA-04**	**37**	**0.35**	
Celebrovascular diseases	VA-04.03	17	0.16	3
Ischemic heart diseases	VA-04.02	10	0.09	8
Congestive Heart Failure	VA-04.05	7	0.07	10
Hypertensive diseases	VA-04.01	3	0.03	
**Perinatal causes of death**	**VA-10**	**24**	**0.23**	
Bacterial sepsis of newborn	VA-10.08	14	0.13	5
Prematurity	VA-10.02	6	0.06	
Congenital malformation of the nervous system	VA-10.09	2	0.02	
Others	VA-10.05, VA-10.06,	2	0.02	
**Neoplasm**	**VA-02**	**20**	**0.19**	
Malignant neoplasm of esophagus	VA-02.02	4	0.04	
Malignant neoplasm of breast	VA-02.08	5	0.05	
Malignant neoplasm of cervix	VA-02.09	4	0.04	
Other	VA-02.05, VA-02. 99, VA-02.10	3	0.03	
Malignant neoplasm of small & large intestine	VA-02.04	2	0.02	
Malignant neoplasm of hyphoid , hematopoietic and related tissue	VA-02.14	2	0.02	
**Mental and nervous system disorders**	**VA-08**	**12**	**0.11**	
Epilepsy	VA-08.02	8	0.08	9
Alzheimer disease	VA-08.01	2	0.02	
Other	VA-08.99, VA-08.96	2	0.02	
**Gastrointestinal disorders**	**VA-06**	**11**	**0.11**	
Acute abdomen	VA-06.06	5	0.05	
Gastric and duodenal ulcer	VA-06.01	3	0.03	
Chronic liver disease	VA-06.02	2	0.02	
Paralytic illeus and intestinal obstruction	VA-06.03	1	0.01	
**Renal disorders**	**VA-07**	**11**	**0.11**	
Renal failure	VA-07.01	11	0.1	7
**Nutritional and endocrine disorders**	**VA-03**	**10**	**0.09**	
Diabetes mellitus	VA-03.03	3	0.03	
Severe malnutrition	VA-03.02	5	0.05	
Other	VA-03.01,VA-03.98	2	0.02	
**Respiratory disorders**	**VA-05**	**3**	**0.03**	
Asthma	VA-05.02	2	0.02	
Other	VA-05.01	1	0.01	
**pregnancy, childbirth and postpartum**	**VA-09**	**3**	**0.03**	
**Indeterminate**	**N/A**	**58**	**0.55**	
**Unspecified causes of death**	**VA-99**	**2**	**0.02**	
**Missing**	**-**	**21**	**0.2**	

Bacterial sepsis was the top killer among infants which caused 30.13 deaths per 1000 person-years. In early (1–4 years) and late (5–14 years) childhoods, severe malnutrition and accidental drowning and submersion were responsible for majority of deaths, respectively. HIV/AIDS and tuberculosis were common causes of death among women of reproductive age (15–49 years). Intestinal and acute lower respiratory tract infections were common causes of death among old age women (65 years and above). Among women of reproductive age, pregnancy and childbirth related causes were responsible for few deaths—3 of 73 deaths (4.1%) or 5.34 deaths per 1,000 person-years (Table 3).

**Table 3:** **Cause and age specific mortality rates (per 1000 person-years) among females in Kilite-Awlaelo health and demographic surveillance system, northern Ethiopia, 2010-2012**

Leading specific causes of death by age group	VA-code	n	Rate
**<1 year** *(person-years = 464.6)*			
Bacterial sepsis of newborn	VA-10.08	14	30.13
Indeterminate	N/A	7	15.07
Prematurity	VA-10.02	6	12.91
Acute lower respiratory infections	VA-01.13	4	8.61
Congenital malformation of the nervous system	VA-10.09	2	4.30
**1-4 years** *(person-years = 4276.8)*			
Severe malnutrition	VA-03.02	4	0.94
Acute lower respiratory infections	VA-01.13	3	0.70
Indeterminate	N/A	3	0.70
Accidental Exposure to smoke, fire and flame	VA-11.05	2	0.47
Intestinal infection diseases	VA-01.01	2	0.47
**5-14 years** *(person-years = 29707.1)*			
Accidental drowning and submersion	VA-11.04	7	0.24
Missing	-	2	0.07
Accidental Fall	VA-11.03	2	0.07
Meningitis	VA-01.11	2	0.07
Tuberculosis	VA-01.03	2	0.07
**15-49 years** *(person-years = 56137.4)*			
Indeterminate	N/A	10	0.18
HIV/AIDS	VA-01.09	8	0.14
Tuberculosis	VA-01.03	8	0.14
Missing	-	5	0.09
Infectious diseases, unspecified cause	VA-01.99	4	0.07
**50-64 years** *(person years = 9758.3)*			
Tuberculosis	VA-01.03	17	1.74
Indeterminate	N/A	6	0.61
Missing	-	4	0.41
Celebrovascular diseases	VA-04.03	4	0.41
Accidental Fall	VA-11.03	2	0.20
**65-84 years** *(person-years = 5075.2)*			
Tuberculosis	VA-01.03	30	5.91
Indeterminate	N/A	21	4.14
Celebrovascular diseases	VA-04.03	11	2.17
Acute lower respiratory infections	VA-01.13	9	1.77
Meningitis	VA-01.11	8	1.58
**85+ years** *(person-years = 374.4)*			
Indeterminate	N/A	10	2.67
Missing	-	5	1.34
Infectious diseases, unspecified cause	VA-01.99	5	1.34
Intestinal infection diseases	VA-01.01	4	1.07
Acute lower respiratory infections	VA-01.13	3	0.80

## Discussion

Although CDs were the leading causes of death in early age group, ECs and NCDs were the most common causes among late childhood and adult females. Particularly NCDs were the most common causes of death among females of 50 years and above. Highly linked to lifestyle, poor eating habit and alcohol consumption, these diseases are becoming increasingly common in the developing countries [3, 4, 25].

Our study population depicts epidemiological transition in which NCDs and injuries becoming common and coexisting alongside CDs among females. Studies in Ethiopia reported that CDs are the most common causes of death among females. At the same time, these studies highlights increasing public health problem of NCDs [3, 4, 26]. This can be attributed to nutritional transitions resulting in obesity which has been found to be increasing in some rural areas in developing countries [27, 28]. Moreover, a report indicated that poor and disadvantaged people are more likely to develop NCDs as a result of the exposures to behavioral risks [29]. Despite the continuing interventions against CDs among female population, these findings highlight public health importance of preventing and controlling of NCDs in developing countries.

Tuberculosis, acute lower respiratory tract infection and intestinal infectious diseases were the leading specific causes of death. Particularly, tuberculosis was the leading cause of death among all female residents. We found also that HIV/AIDS and tuberculosis were the major causes of death among women of reproductive age. This implies the need for at least equal attention for other causes of death like HIV/AIDS and tuberculosis among women of reproductive age group in addition to maternal causes. According to the 2013 global tuberculosis report, Africa is one of the regions with slow pace to achieve the mortality elated to tuberculosis. According to the report, tuberculosis remains among the top three killers of women [30]. In line with our findings, different studies have also reported the public health burden of tuberculosis and HIV/AIDS among female population in sub-Saharan African countries including Ethiopia [2, 4, 11, 31–33]. These finds signal the need of more efforts to prevent tuberculosis related mortality among females.

Consistent with the epidemiological trend of mortality in developing countries, higher mortality rates were recorded among old age groups (106.84 per 1000 person-years) and infants (101.16 per 1000 person-years). This is expected as elders and infants have weak and immature immune system to defend diseases. Furthermore, these population groups are prone for variety of diseases like congenital abnormalities and chronic diseases. The leading causes of death among infants were perinatal causes and this is in line with other study in Ethiopia [3]. In concurrence with other studies in Ethiopia [3, 4, 10], pregnancy and childbirth related causes were responsible for few deaths, causing only 3 deaths (4.1%) or 5.34 deaths per 1000 person-years among women of reproductive age.

Our study had some limitations. We had 21 (5.3%) cases whose were their verbal autopsy data were not complete. Hence, they were labeled as “missing” causes of death which could have had important implications on rates of causes of death if they had been correctly collected and assigned. On top of this, the performance related to VA questionnaires and physicians in detecting exact causes of death for a given case could be mentioned as a limitation. It is also important to note that interpreting the VAs and assigning causes of death by physicians have been questioned for its reliability and repeatability [34].

## Conclusions

In summary, we got mortality information coupled with VA data which allowed us to estimate cause- and age-specific mortality among female residents in KA-HDSS site. Despite the continuing public health burden of CDs, NCDs and ECs were significantly contributing for female deaths. Tuberculosis, cerebrovascular diseases and accidental falls were the leading specific causes of deaths under the categories of CDs, NCDs and ECs, respectively. CDs are continued to be the leading causes of death among females. HIV/AIDS and tuberculosis were the major causes of death among women of reproductive age.

Despite the attention given and efforts being made to prevent maternal causes of death in Ethiopia, other causes of female deaths are equally important to consider in maternal health related interventions. In rural part of Ethiopia, existing public health and curative interventions on CDs should be strengthened. Alongside with the existing programs and strategies to expand maternal health care services, the Ethiopian Ministry of Health and Tigray regional Health Bureau should scale up the current efforts to reverse the public health burden of other causes of death, like HIV/AIDS and tuberculosis, among females.

## Authors’ information

Authors are research team members of Kilite-Alaelo Health and Demographic Surveillance System (KA-HDSS) under Mekelle University, College of Health Sciences. All are interested in health and medical researches.
